# Utility of inhaled β2-agonists in reducing serum potassium levels in adult patients with hyperkalemia: A scoping review

**DOI:** 10.1371/journal.pone.0342309

**Published:** 2026-02-03

**Authors:** Leonardo Arzayus-Patiño, Ashley Y. Hinojosa-Angulo, Karen A. Rodríguez-Angulo, Michelle A. Sánchez-Caicedo, Vicente Benavides-Córdoba

**Affiliations:** 1 programa de Fisioterapia, Facultad de Salud, Universidad Santiago de Cali, Cali, Colombia. Grupo de Investigación Salud y Movimiento, Facultad de Salud, Universidad Santiago de Cali, Cali, Colombia; 2 Facultad de Salud, Universidad del Valle, Cali, Colombia; Stellenbosch University Faculty of Medicine and Health Sciences, SOUTH AFRICA

## Abstract

**Introduction:**

Hyperkalemia is a potentially life-threatening condition that requires prompt intervention to prevent cardiac complications. While insulin and glucose administration remains a cornerstone of treatment, inhaled β2-adrenergic agonists have been proposed as a complementary or alternative strategy, particularly in emergency settings. This scoping review aimed to describe the utility of inhaled β2-agonists in lowering serum potassium levels in adult patients.

**Methods:**

A scoping review was conducted following the PRISMA-ScR guidelines and the Joanna Briggs Institute methodology. Experimental studies published within the past ten years evaluating the use of inhaled β2-agonists in adult patients with hyperkalemia were included. Five studies were analyzed (three randomized controlled trials and three quasi-experimental studies), assessing dosage, route of administration, magnitude of potassium reduction, and reported adverse events.

**Results:**

Most studies used nebulized salbutamol at a dose of 10 mg, with observed reductions in serum potassium ranging from 0.62 to 1.636 mEq/L, and a peak effect between 1 and 4 hours post-administration. One study also reported the use of levalbuterol. The most common adverse effects were tachycardia, dizziness, and mild hyperglycemia, all of which were clinically manageable. Efficacy was demonstrated in both patients with chronic kidney disease and in individuals with normal renal function.

**Conclusion:**

Inhaled β2-agonists, particularly nebulized salbutamol, represent an effective and safe therapeutic option for the acute reduction of serum potassium in adults with hyperkalemia. Their rapid onset of action and applicability across various patient profiles make them a valuable tool in emergency settings, especially where immediate access to advanced therapies such as dialysis is limited. Further research is warranted to evaluate long-term outcomes, safety in patients with cardiovascular comorbidities, and optimal dosing strategies.

## Introduction

Potassium is a key electrolyte responsible for proper cellular function, particularly in muscles and nerves. It plays a crucial role in regulating heart rhythm, muscle contraction, and nerve impulse transmission. Additionally, the kidneys play a vital role in maintaining potassium homeostasis, regulating its excretion through urine

Both hypokalemia and hyperkalemia can have serious health consequences, including cardiac arrhythmias, muscle weakness, and fatigue. This imbalance disrupts the electrical stability of cells, especially in the myocardium, potentially leading to life-threatening arrhythmias and even cardiac arrest if not promptly treated. Potassium plays a fundamental role in maintaining the resting membrane potential of excitable cells, including cardiac conduction tissue and skeletal muscle fibers, thereby preserving normal electrical signaling [1,2].

Hyperkalemia is defined as a serum potassium concentration above the normal range, typically exceeding 5.0 to 5.5 mEq/L [3]. Its critical role in cellular repolarization is mediated by the sodium-potassium ATPase pump, meaning that any alteration in potassium levels can significantly impair this process, increasing the risk arrhythmias and cardiac arrest [4].

The most common cause of hyperkalemia in adults is impaired renal potassium excretion, most often resulting from chronic kidney disease or acute kidney injury. When renal excretion of excess potassium is impaired, potassium accumulates in the bloodstream certain medications including angiotensin-converting enzyme inhibitors (ACEIs), potassium-sparing diuretics, and nonsteroidal anti-inflammatory drugs (NSAIDs) may also contribute to the development of hyperkalemia [4].

Additional risk factors include poorly controlled diabetes mellitus, adrenal insufficiency, and conditions associated with massive cellular breakdown, crush injury or rhabdomyolysis [5]. Early identification and prompt treatment of hyperkalemia are critical to preventing serious complications. Rapid correction is essential to avoid arrhythmia and cardiac arrest [5].

Despite its high prevalence and clinical significance, management of hyperkalemia varies across clinical guidelines, which differ in thresholds for intervention, preferred pharmacologic agents, and strategies for urgent versus definitive management [6]. Patients receiving renin-angiotensin-aldosterone system inhibitors or mineralocorticoid receptor antagonists, key therapies in cardiovascular disease are particularly vulnerable, with many experiencing recurrent hyperkalemia. This often leads to dose reduction or discontinuation, potentially compromising cardiovascular outcomes [1,4]. Management in adults requires a multidisciplinary approach focused on identifying and treating the underlying cause while implementing immediate interventions to lower serum potassium levels and prevent cardiovascular complications. Continuous electrocardiographic (ECG) monitoring is essential to detect and manage potentially life-threatening arrhythmia [5].

Initial treatment often includes intravenous calcium gluconate, which, although it does not reduce serum potassium levels, stabilizes the cardiac membrane and provides protection against arrhythmias resulting from excessive repolarization. Concurrently, agents such as insulin with glucose are administered to promote the intracellular shift of potassium, temporarily lowering its serum concentration. Potassium-binding resins, such as sodium polystyrene sulfonate, aid in intestinal elimination of potassium. In severe cases, hemodialysis remains the most effective and rapid method for significantly lowering serum potassium levels [6]. Finally and the focus of this review—β2-adrenergic agonists have also been proposed for this purpose.

β2-adrenergic agonists, commonly used to manage bronchospasm in respiratory conditions, exert their effects by activating Gs protein-coupled β2-adrenergic receptors, stimulating adenylate cyclase, and increasing cyclic AMP (cAMP) production from ATP. cAMP activates protein kinase A (PKA), which phosphorylates and stimulates Na ⁺ /K ⁺ -ATPase activity, thereby enhancing potassium uptake into cells. Additionally, PKA modulates voltage-gated potassium channels, reducing potassium efflux and promoting intracellular redistribution [7]. Through this mechanism, β2-agonists have been repurposed as adjunctive agents in hyperkalemia management, aligning with the drug repurposing strategy [8]. Their administration, typically via inhalation, is theoretically a practical and accessible therapeutic option [9].

Although β₂-adrenergic agonists are generally well tolerated, they should be used with caution in patients with underlying cardiac conditions, as they may increase heart rate and produce other side effects due to β₁-receptor activation in the myocardium. This effect is likely related to the higher doses required to achieve potassium lowering, which are often two to four times greater than those used for the treatment of bronchospasm [9].

The potential benefit of β2-agonists in the management of hyperkalemia has been explored in several studies [10]. However, uncertainty remains regarding whether inhaled administration of these agents consistently produces a clinically meaningful reduction in serum potassium, and the optimal dose and frequency of administration are yet to be established [10]. In Colombia, limited access to specialized treatments such as hemodialysis especially in rural areas due to geographic constraints underscores the importance of identifying alternative initial management strategies. Early administration of inhaled β2-agonists may provide a temporizing measure to stabilize patients while definitive treatment is arranged. Although this approach may not match the efficacy of hemodialysis in correcting severe hyperkalemia, it may buy critical time and reduce the risk of fatal cardiac complications, potentially improving survival in resource-limited settings [1].

The objective of this review is to map and describe the current literature on short-acting inhaled β2-adrenergic agonists in adult patients with hyperkalemia, focusing on their effects in reducing serum or plasma potassium levels, and to identify existing evidence gaps.

## Methods

This scoping review was conducted following the guidelines outlined in the PRISMA-ScR (Preferred Reporting Items for Systematic Reviews and Meta-Analyses extension for Scoping Reviews) checklist, ensuring methodological transparency and rigor. In addition, the methodological framework proposed by the Joanna Briggs Institute (JBI) was applied, which includes the following phases: formulation of the research question, identification of relevant studies, study selection, data extraction, synthesis of findings, and reporting of results and conclusions.

### Research question

To guide the scoping review, the following research question was formulated:

What is the existing evidence on the use of short-acting inhaled β2-adrenergic agonists in adult patients with hyperkalemia?

The question was structured based on the PCC framework:

P (Population): Adult patients with hyperkalemia

C (Concept): Use of short-acting β2-adrenergic agonists

C (Context): Clinical management of hyperkalemia

### Selection criteria

#### Inclusion criteria.

To identify relevant studies, a literature search was conducted based on the research question. Studies were selected if they met the following inclusion criteria:

- Primary research articles, systematic reviews, expert recommendations, gray literature, and both qualitative and quantitative studies
- No restriction on publication date
- Publications in English, Spanish, or Portuguese
- Studies, published or unpublished, that addressed the research question in a relevant manner

#### Exclusion criteria.

Studies in which short-acting β₂-adrenergic agonists were administered alongside co-treatments, such as insulin–glucose or potassium-binding agents, were excluded when the independent effect of the β₂-agonist on serum potassium could not be determined.

### Information sources

Based on the research question, a comprehensive literature search was conducted in the following electronic databases: PubMed, Google Scholar, Scopus, MEDLINE, and SciELO. Relevant keywords and standardized descriptors were defined using Medical Subject Headings (MeSH) and Descritores em Ciências da Saúde (DeCS). Reference lists from included articles were also reviewed to identify additional relevant sources.

### Search strategy

The search strategy combined controlled vocabulary and free-text terms. Standardized descriptors such as MeSH and DeCS were employed, together with specific keywords in English, Spanish, and Portuguese, to construct precise search strings tailored to each database. Keywords were organized according to the PIO structure (Population, Intervention, Outcome), as shown in Table 1, which facilitated the development of search equations based on thesauri descriptors and qualifiers. The specific search strings applied in each database are detailed in Table 2.

**Table 1:** DeCS – MESH.

	*DeCS*	*MESH*
***P***	*Adulto, adultos*	*Adult*
***C***	*Efecto Broncodilatador Broncodilatadores, agentes broncodilatadores, salbutamol o albuterol.*	*Effect, Bronchodilator, Bronchodilator Agent, Broncholytic Agent, Adrenergic beta-2 Receptor Agonists, beta-2 Agonists, Adrenergic, Adrenergic beta2-Agonists, terbutaline, levalbuterol, albuterol*
***C***	*Potasio, Potássio DISMINUCIÓN, NORMALIZACIÓN POTASIO, Hiperkalemia, hiperpotasemia*	*Potassium* *Hyperkalemia* *Hyperpotassemia*

*Source: Own elaboration.*

**Table 2:** Databases searched, search strategies, and study selection process.

	Acronym					Search
Database	Number of Articles	ST	AS	FR	FS	Search Equation
**Pubmed**	134	34	10	1	1	(Hyperkalemia) AND (Receptor Agonists Albuterol OR salbutamol)
**Google scholar**	233	100	50	6	1	Adrenergic beta-2 Receptor Agonists AND Hyperpotassemia AND Hyperkalemia therapy
**Scopus**	315	8	3	2	3	(“Adrenergic beta-2 Receptor Agonists AND Albuterol AND Hyperkalemia therapy”)
**Medline plus**	0	0	0	0	0	(“Broncodilatadores beta-2 adrenérgicos”OR “Agonistas beta-2 adrenérgicos” AND (“Hiperpotasemia”) AND (“Adult”)
**SCIELO**	1	0	0	0	0	(albuterol OR salbutamol) AND (hiperpotasemia OR hyperkalemia) AND (tratamiento OR intervencion)

Source: Authors’ own elaboration. ***ST:*** *selected by title;* ***AS:*** *abstract selected;* ***FR:*** *full reading;* ***FS:*** *final selection*

This comprehensive approach enhanced both the accuracy and completeness of study identification. To ensure thoroughness, the automated search was complemented by a manual review of the reference lists of the included studies, as well as an advanced search using additional keywords. The literature search was conducted between September 2024 and March 20*DeCS – MESH*25, with the last update performed in March 2025.

To initiate the search, concepts were standardized using the databases available through the Virtual Health Library (VHL) portal and PubMed. Subsequently, an advanced bibliographic search was conducted in the databases listed in Table 2, using DeCS/MeSH terms in English, Spanish, and Portuguese, and a specific search equation tailored to each database.

### Selection of sources of evidence

The search was conducted by three researchers. The processes of identification, screening, and eligibility assessment were carried out through consensus among the investigators. A fourth researcher was involved to verify the effectiveness of the search strategy and to resolve any discrepancies.

### Data extraction

Once the articles were identified, the researchers performed a critical reading and compiled a descriptive table with the relevant data from each study. One team member analyzed and reviewed the articles to confirm their relevance and compliance with the inclusion criteria. No discrepancies were reported among the reviewers during this process. Table 3 presents the characteristics of each included study, including author, year, country, objective, population and sample size, study design, evaluated variables, dosage and frequency, medication and type, results, and conclusion. Duplicate articles were removed, and additional studies were filtered based on the information provided.

**Table 3:** Characteristics of the studies.

	Study Objective	Population and Sample	Study Design	Evaluated Variables	Device	Dose/Frequency	Medication	Results	Conclusion
Avigdor Mandelberg et al/ United States/ 1999 (13)	To determine the efficacy of inhaled salbutamol in reducing serum potassium levels in patients with hyperkalemia.	**Population:** 17 patients with chronic kidney disease on hemodialysis, randomly and blindly assigned to two groups. **Group 1:** received salbutamol before dialysis, then placebo before the next session. **Group 2:** received placebo first, followed by salbutamol.	Randomized, double-blind, placebo-controlled clinical trial.	• Reduction in serum potassium levels after salbutamol inhalation compared to placebo. • Onset time of the hypokalemic effect following drug administration. • Repeated monitoring of heart rate and blood pressure. • Measurement of insulin levels in a subset of patients (n = 10) before, and at 1 and 5 minutes after inhalation.	Metered-dose inhaler (MDI)	Each patient inhaled 1,200 micrograms of salbutamol using a metered-dose inhaler with spacer (MDI-S) over a 2-minute period. Blood samples were collected repeatedly before administration and at 1, 3, 5, 10, and 60 minutes after inhalation.	Salbutamol	A transient increase in serum potassium was observed one minute after salbutamol inhalation, followed by a progressive decline. During the treatment period, 10 out of 17 patients (59%) experienced an increase of ≥ 0.1 mEq/L, while no changes were observed during the placebo period (0%) (p < 0.0001). From three minutes post-inhalation, potassium levels decreased significantly over time, in contrast to the placebo group, where no time-dependent variations were detected (p < 0.001). The difference between the placebo and salbutamol treatment periods reached statistical significance at five minutes (p < 0.05). Additionally, a significant increase in serum glucose was observed at three minutes after salbutamol inhalation. Heart rate also increased significantly within the first five minutes. As for serum insulin, no changes were noted at one minute post-inhalation; however, a significant elevation was detected at five minutes.	The inhalation of 1,200 micrograms of salbutamol via a metered-dose inhaler exhibits a relatively rapid onset of action, inducing a progressive and sustained reduction in serum potassium levels beginning between 3 and 5 minutes after administration.
N. Karuna Sree and R. et al/ India/ 2011 (15)	To evaluate the efficacy of salbutamol nebulization in reducing serum potassium levels in patients with hyperkalemia associated with renal failure.	30 patients with acute severe renal failure and hyperkalemia (serum potassium > 5.5 mEq/L).	Open-label study.	Serum potassium levels (at 0, 1, 4, and 6 hours post-nebulization), electrocardiogram (ECG) before and after treatment, serum glucose, blood urea, and creatinine.	Jet nebulizer device	10 mg of salbutamol nebulized over 10 minutes using a face mask.	salbutamol	A reduction in serum potassium was observed in all patients except two, who were classified as non-responders. The average decrease was 1.636 mEq/L, with the maximum effect occurring within the first 4 hours. Additionally, electrocardiographic abnormalities were normalized.	Salbutamol nebulization is an effective and safe intervention for the rapid reduction of serum potassium in patients with hyperkalemia secondary to renal failure. It can be used as an initial treatment or in combination with other therapies until hemodialysis becomes available.
Michael Allon, MD et al/ United States/ 1989 (16)	To determine the efficacy and safety of albuterol nebulization in the acute treatment of hyperkalemia in patients undergoing chronic hemodialysis.	0 hemodialysis patients with chronic hyperkalemia.	Prospective, double-blind, placebo-controlled study.	Plasma potassium concentration at multiple time points (0, 30, 60, and 120 minutes), blood pressure, and heart rate.	Jet nebulizer device	Nebulization of albuterol (10 mg or 20 mg) or placebo (saline solution), administered on three separate occasions. Parameters were monitored for 2 hours after administration.	Albuterol	A significant reduction in plasma potassium was observed at 30 minutes and was sustained for at least 2 hours. The mean decrease was 0.62 ± 0.09 mEq/L with 10 mg of albuterol and 0.98 ± 0.14 mEq/L with 20 mg. Placebo had no effect on potassium concentration. No significant cardiovascular adverse effects were reported.	Nebulization of 10 mg albuterol is an effective and safe option for the acute treatment of hyperkalemia in hemodialysis patients, with no significant cardiovascular adverse effects.
Hung-Hsiang Liou et al./ Taiwan and United States/ 1994 (17)	To compare the efficacy and safety of intravenous versus nebulized salbutamol administration in the treatment of hyperkalemia in patients with chronic renal failure.	15 patients with chronic renal failure, with blood urea nitrogen levels > 80 mg/dL and creatinine > 8.0 mg/dL.	Quasi-experimental (comparative study).	Plasma potassium levels at multiple time points (0, 10, 20, 30, 60, 90, 120, and 180 minutes), along with insulin, glucose, sodium, osmolality, pH, blood pressure, and heart rate.	Jet nebulizer device	Administration of two treatment modalities at separate times: intravenous infusion of 0.5 mg salbutamol in 100 mL of 5% dextrose over 15 minutes, and nebulization of 10 mg salbutamol in 5 mL of saline solution over 15 minutes.	Salbutamol	Both intravenous infusion and nebulization of salbutamol significantly reduced plasma potassium levels. The maximum reduction was similar for both routes: 0.92 ± 0.10 mEq/L for intravenous administration (maximum effect at 30 minutes) and 0.85 ± 0.13 mEq/L for nebulization (maximum effect at 90 minutes). Nebulization exhibited a more prolonged effect, maintaining a significant potassium reduction up to 180 minutes. Increases in insulin and glucose levels were observed with both methods. Heart rate increased in both groups, though to a lesser extent with nebulization. No significant changes were noted in blood pressure, pH, or sodium levels.	Both intravenous infusion and nebulization of salbutamol are effective and safe treatments for hyperkalemia in patients with chronic renal failure. Intravenous administration provides a faster response and is recommended in urgent situations, whereas nebulization, due to its lower cardiovascular impact, may be preferable for patients with underlying heart disease.
Martha Jeanette Rojas García/ Mexico/ 2010 (18))	To determine, evaluate, and quantify the efficacy of intravenous salbutamol versus salbutamol administered via micronebulizer in the treatment of acute hyperkalemia in patients with chronic kidney disease not receiving renal replacement therapy.	62 adult patients (aged 18–90 years) with chronic kidney disease not receiving renal replacement therapy and with serum potassium levels ≥ 5.5 mEq/L, admitted to the emergency department. The patients were divided into two groups of 31 participants each.	Randomized clinical trial.	Serum potassium levels before and 60 minutes after treatment. Adverse effects such as tachycardia, headache, and dizziness were documented.	Jet nebulizer device	• **Intravenous group:** 0.5 mg of salbutamol diluted in 100 mL of 5% dextrose solution, infused over 15 minutes. • **Micronebulization group:** 10 mg of salbutamol diluted in 2 mL of injectable solution, administered via face mask over 15 minutes.	Salbutamol	Both routes of administration significantly reduced serum potassium levels (p < 0.004). There was no statistically significant difference between the post-treatment means of the two groups (p = 0.1). Regarding adverse effects, tachycardia was reported in 21.2% of patients (12.1% in the nebulization group and 9.1% in the intravenous group). Headache was observed only in 3% of the intravenous group, while dizziness was reported in 7.6% of cases (4.5% in nebulization and 3.1% in intravenous administration).	The administration of salbutamol is an effective and safe strategy for lowering serum potassium in patients with chronic kidney disease not undergoing replacement therapy. No significant difference in efficacy was observed between the intravenous and nebulized routes; however, tachycardia was more frequently reported in the nebulization group..

### Critical appraisal

Risk of bias was assessed using two specific tools according to the study design. For randomized controlled trials, the ROB 2 (Risk of Bias 2.0) tool was used, which is designed to evaluate bias in studies with random allocation of participants. For single-group experimental studies, the Joanna Briggs Institute (JBI) checklist for before-and-after studies without control groups was applied. Each tool allows for the identification and categorization of potential biases across various domains, ensuring a structured and rigorous analysis of the methodological validity of the included studies.

### Presentation of results

**Fig 1:**
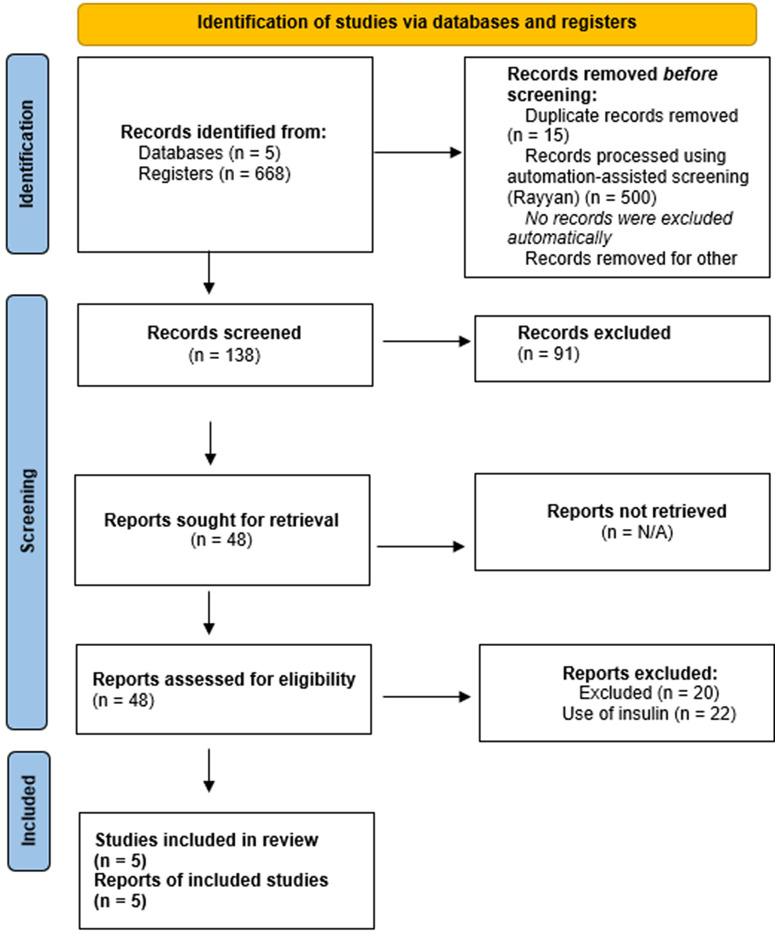
Flow diagram of the literature search according to PRISMA guidelines.

The results are presented in descriptive tables outlining key aspects of each study, such as author, study objective, population, sample size, medication used, dosage, observed effect, main outcomes, and conclusions. Additional tables summarize the search strategy, including the search equations used (Table 3), and the final number of studies included. A flow diagram (Fig 1) illustrates the search process and final number of studies retained for analysis.

### Ethical statement

As this is a scoping review, ethical approval is not required for this type of study. No primary data was collected from participants, and all studies included in this review were publicly available.

## Results

A total of 668 studies were identified. After initial screening by title and abstract, inclusion and exclusion criteria were applied, leading to the exclusion of 659 studies. Ultimately, 5 articles were included in the review (Fig 1).

All included studies were experimental in design. Three were randomized controlled trials (RCTs) that compared the use of inhaled β2-agonists with either placebo or intravenous administration [11,12], while the remaining three were quasi-experimental studies without control groups [13–15]. All of them evaluated the effectiveness of β2-agonists in reducing serum potassium levels.

The included studies evaluated the intervention of inhaled β2-agonists in a total of 161 patients for the management of hyperkalemia. The most commonly used drug was salbutamol; however, one study included the use of levalbuterol. No studies reported the use of other β2-agonists such as fenoterol or terbutaline. Regarding the mode of administration, nebulization was the predominant method, with only one study reporting the use of a metered-dose inhaler (MDI) [11].

The most frequently reported dose of nebulized salbutamol in RCTs was 10 mg, although one study used a higher dose of 20 mg. In the study using MDI, a single dose of 1,200 mcg was administered [11].

The analyzed studies showed that β2-agonists effectively reduced serum potassium levels, with variations in the magnitude and duration of the effect depending on the dose and route of administration. In general, the reduction in potassium was observed between 5 and 30 minutes after administration, with the peak effect occurring between 1 and 4 hours. Nebulization may offer an effective route, with the available studies reporting sustained reductions in potassium concentration (studies 5 and 6) [12,15]. A mean decrease ranging from 0.62 to 0.98 mEq/L was reported with 10–20 mg of nebulized albuterol (study 4) [14], and up to 1.636 mEq/L in responsive patients (study 3) [13]. In contrast, the study using 1,200 mcg via MDI reported a transient increase in potassium at the onset of treatment, followed by a progressive decline (study 1) [11].

Across the included studies, adverse events were generally mild and occurred at low frequency when percentages were reported. Rojas García et al. documented tachycardia in 12.1% of patients treated via micronebulization and in 9.1% of those receiving intravenous administration, as well as headache (3%) and dizziness (4.5% in the nebulization group and 3.1% in the intravenous group). Regarding non-responders, Karuna Sree et al. identified two patients who did not exhibit a reduction in serum potassium, while Liou et al. reported a non-response rate of 33.3% in the intravenous group (5/15), with full response in the nebulized group. The remaining studies did not quantify non-response, although they consistently reported overall reductions in serum potassium levels [11,14,15].

The duration of the hypokalemic effect of β2-agonists was evaluated, with one study showing a sustained reduction in potassium for at least 2 hours [14], another reporting a prolonged effect up to 180 minutes [15], and another documenting peak efficacy within the first 4 hours [13].

To facilitate comparison between studies, a summary table (Table 4) was added that highlights key patterns related to dose, route of administration, magnitude of potassium reduction, time to maximum effect, and reported safety outcomes, as well as available information on non-responder patients.

**Table 4:** Overview of dosage, efficacy, and safety profiles of β₂-agonists.

Study	Dose & Delivery Route	Mean Potassium Reduction	Time to Peak Effect	Reported Adverse Events	Non-Responders
**Mandelberg et al., 1999**	1,200 mcg salbutamol (MDI + spacer)	Progressive decline after initial transient rise	3–5 min onset; peak ≈ 60 min	Palpitations, tachycardia, tremor, headache, transient increase in glucose and heart rate	Not found
**Karuna Sree et al., 2011**	10 mg salbutamol (nebulized)	Mean ↓ 1.636 mEq/L	Within 4 h	No reported cardiovascular adverse effects or significant symptoms	Not found
**Allon et al., 1989**	10 mg or 20 mg albuterol (nebulized)	↓ 0.62 to 0.98 mEq/L	30 min; sustained up to 2 h	Heart rate increase, insulin and blood glucose rise; no changes in BP, pH, Pco₂, sodium, or osmolality	IV: 5/15 (33.3%) non-responders; Neb: 0/10
**Liou et al., 1994**	0.5 mg IV or 10 mg nebulized	↓ 0.92 mEq/L (IV) and 0.85 mEq/L (neb)	30 min (IV), 90 min (neb)	Tachycardia (12.1% Neb; 9.1% IV), headache (3% IV, 0% Neb), dizziness (4.5% Neb; 3.1% IV)	Not found
**Rojas García, 2010**	0.5 mg IV vs. 10 mg nebulized	Significant reduction (no difference between groups)	60 min	Palpitations, tachycardia, tremor, headache, transient increase in glucose and heart rate	

**Fig 2:**
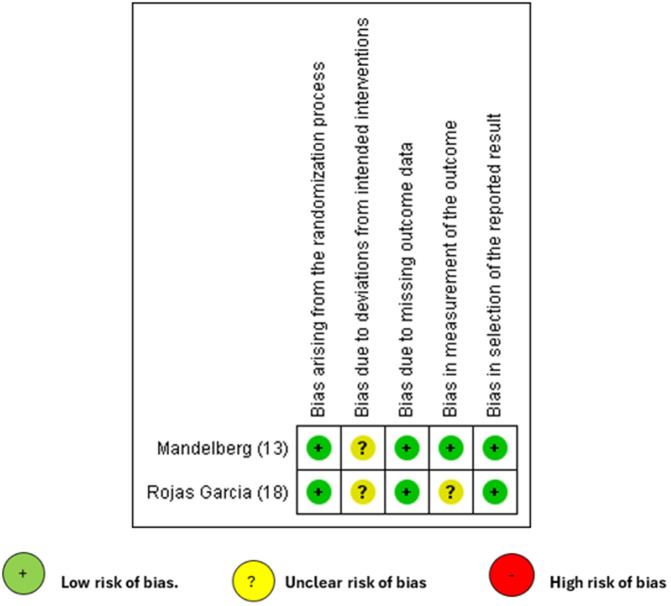
Quality of the studies.

The risk of bias was assessed using the ROB 2 tool. All three RCTs showed low risk in most domains, with only one domain showing some concerns, indicating high methodological quality with a mild degree of uncertainty Fig 2. The quasi-experimental studies evaluated using the Joanna Briggs Institute checklist demonstrated moderate to high methodological quality, reflecting rigor in both design and execution Table 5.

**Table 5:** Quality of the studies.

Author	Study Type	Quality Assessment (Tool-Evaluation)	Interpretation
N. KARUNA SREE 20	Single-group experimental study	Joanna Briggs Institute, score:63%	Moderate quality
Liou et al. (1994) #16	Single-group experimental study	Joanna Briggs Institute, score: 86%	High quality.
Allon et al. (1989 #15)	Single-group experimental study	Joanna Briggs Institute, score:: 100%	High quality.

## Discussion

The objective of this review was to describe and analyze the available evidence regarding the benefits of β2-agonist administration in the management of hyperkalemia in adult patients. The findings suggest that inhaled β2-agonists may help reduce serum potassium over a short period of time, although the limited number of studies and small sample sizes mean that these results should be interpreted cautiously. The magnitude and duration of the effect vary across studies, raising questions about the factors that influence their efficacy.

These results highlight the need to further explore the impact of dosage, route of administration, and patient characteristics on treatment response. The reduction in serum potassium following β₂-agonist administration is primarily mediated by stimulation of β₂-adrenergic receptors in skeletal muscle, leading to activation of the Na ⁺ /K ⁺ -ATPase pump and subsequent intracellular shift of potassium [9]. This transcellular redistribution contributes to membrane repolarization and explains the hypokalemic effect observed with β₂-agonists. This mechanism has been supported by previous studies and by the consensus of the Spanish Society of Nephrology, which recommends β₂-adrenergic agonists as a strategy for managing hyperkalemia and emphasizes their role in regulating transcellular potassium movement, particularly when combined with other agents such as insulin to enhance therapeutic efficacy [16].

Among the β2-agonists used for lowering serum potassium, the reviewed studies exclusively mentioned salbutamol (also known as albuterol, depending on the region) and levalbuterol, a purified form of salbutamol. However, levalbuterol current evidence does not suggest meaningful superiority to salbutamol in terms of clinical efficacy or safety, although it is associated with a higher cost [17,18]. Therefore, its cost-effectiveness favors the implementation of salbutamol in clinical settings.

No clinical studies were found evaluating potassium reduction with other short-acting β2-adrenergic agonists such as fenoterol or terbutaline, which limits the ability to reliably extrapolate the hypokalemic effects of these agents. While some studies have suggested that fenoterol may have greater bronchodilator potency, it also exhibits lower β2-receptor selectivity, potentially increasing adverse effects related to β1-receptor activation in the heart, such as tachycardia and palpitations [19,20]. Although the hypokalemic mechanism of action may be similar across different β2-agonists, the absence of specific clinical evidence, limits the ability to determine equivalence, in efficacy and safety for hyperkalemia management.

Most studies included in this review used jet nebulizers, with only one study reporting the use of a metered-dose inhaler (MDI). This contrasts with routine respiratory practice, where MDIs are more prevalent. Although some studies have shown that MDIs with spacers may achieve effective pulmonary drug deposition at lower doses of salbutamol in the management of acute bronchospasm [21], a systematic review reported no significant differences in clinical effectiveness between MDIs and nebulizers in patients with COPD [22]. In the context of hyperkalemia management where patients typically do not present with acute respiratory failure the choice of delivery method appears to be influenced more by practicality, availability, and ease of use than by demonstrated differences in potassium-lowering efficacy.

In the reviewed studies, the doses of β2-agonists used to lower serum potassium were higher than those typically employed for respiratory conditions. This reflects the need for a more pronounced and rapid hypokalemic effect in the management of hyperkalemia, while highlighting that direct comparisons between delivery methods should be interpreted cautiously due to differences in drug administration efficiency [23–25].

Although in the context of respiratory crises the frequency of administration is usually higher, with intervals of only a few minutes between doses, the studies in the hyperkalemia literature generally evaluate a single administration. Thus, it cannot be concluded that both routes are equivalent in terms of a single dose, which may justify the use of higher drug doses. However, this difference in dosing underscores the need for caution when comparing equivalence between nebulization and MDI delivery.

It is not appropriate to directly extrapolate a 10 mg dose of nebulized salbutamol to its equivalent via metered-dose inhaler (MDI), as this would require 10,000 mcg (100 inhalations), considering that commercial devices typically deliver 100 mcg per actuation. The discrepancy in required dosing between both delivery systems may be attributed to differences in drug delivery efficiency: in nebulization, a significant portion of the drug is not effectively inhaled due to factors such as the patient’s respiratory pattern, variability in inspiratory and expiratory times, and the flow rate when administered in combination with supplemental oxygen [26]. Therefore, there is currently no evidence to support the recommendation of doses greater than 1,200 mcg via MDI for potassium-lowering purposes, whereas 10 mg via nebulization remains the most widely used and documented dose in the available literature.

When analyzing the reduction in serum potassium, this finding is clinically meaningful, as it may help prevent severe complications associated with hyperkalemia, particularly cardiac electrical disturbances that impair effective myocardial contraction. One study in particular demonstrated not only a decrease in serum potassium levels but also normalization of electrocardiographic abnormalities, suggesting restoration of cardiac electrical activity [27]. Importantly, these transient and reversible improvements in cardiac conduction may provide critical time for healthcare teams to stabilize the patient, address the underlying cause most commonly renal dysfunction, with or without impaired potassium elimination due to medications and initiate definitive management, including transfer to centers where advanced therapies such as dialysis are available [15,27,28].

These findings align with previous studies, such as those by Allon et al., which recommend the combined use of β₂-agonists with insulin and glucose as part of so-called polarizing therapies. This combination exerts a synergistic effect by enhancing the intracellular shift of potassium through complementary activation of the Na ⁺ /K ⁺ -ATPase, thereby improving acute control of hyperkalemia. Despite these positive effects, β₂-agonists are not widely adopted in routine practice, likely due to concerns regarding cardiovascular safety, variability in guideline recommendations, and the lack of recent high-quality clinical trials.

From a clinical standpoint, the relatively rapid onset and moderate duration of the hypokalemic effect observed with β₂-agonists support their role as a temporizing measure in the acute management of hyperkalemia. The potassium-lowering effect, which has been reported to persist for approximately 2–4 hours on average, may allow clinicians to stabilize patients while diagnostic evaluation is completed and definitive therapies are arranged. This temporal profile is particularly relevant in emergency and resource-limited settings, including rural areas, where immediate access to renal replacement therapy may not be available. In such scenarios, β₂-agonists can provide a temporary stabilizing effect, reducing the risk of life-threatening complications and allowing critical time for the initiation of definitive treatment [11–15,27].

In comparison with other studies in the literature, there is consistency regarding the rapid onset and moderate duration of the hypokalemic effect of β₂-agonists, reinforcing their usefulness in the acute management of hyperkalemia. However, it is important to consider individual patient characteristics and potential contraindications, such as unstable angina or acute myocardial infarction, in which the use of β₂-agonists is contraindicated. Additionally, studies conducted in healthy individuals, such as that by Pancu et al. [27]., provide complementary evidence supporting the previously described mechanism. Their findings demonstrated a potassium-lowering effect following β₂-agonist administration even in individuals without hyperkalemia, reinforcing that the transcellular shift induced by β₂-receptor activation is a consistent physiological phenomenon. Although these results are not directly generalizable to clinical populations, they contribute to understanding the biological basis underlying the therapeutic use of these agents.

In terms of safety, the reviewed studies reported adverse effects such as tachycardia, headache, dizziness, and elevated blood glucose levels [13–15]. These events may be explained by cross-activation of cardiac β₁-receptors, despite the fact that the β₂-adrenergic agents used have predominant selectivity for pulmonary β₂-receptors [29,30]. Theoretically, this activation may primarily induce tachycardia as well as symptoms related to peripheral vasodilation, such as headache and dizziness. Additionally, blood glucose elevation is associated with β₂-receptor stimulation in extrapulmonary tissues, particularly in the liver, where it increases glycogenolysis and gluconeogenesis, leading to transient hyperglycemia. Similar results have been documented in studies involving patients with diabetes, showing mild and transient increases in blood glucose following β₂-agonist administration, although these changes were not considered clinically relevant. In this context, the modest hyperglycemic effect of β₂-agonists may be clinically relevant when these agents are co-administered with insulin and dextrose, as it could theoretically mitigate the risk of hypoglycemia a well-recognized complication of insulin-based therapy for hyperkalemia. However, this potential benefit has not been systematically evaluated and should therefore be interpreted with caution.

Other commonly reported adverse effects include muscle tremors, anxiety, and muscle cramps. These side effects are generally transient and tend to diminish with dose adjustment or continued exposure. However, they should be monitored, especially in patients with pre-existing conditions such as cardiovascular disease or diabetes [26,31]. Overall, in the included studies, these side effects were temporary, clinically manageable, and not associated with serious complications or major clinical events. Importantly, no cases of myocardial ischemia or acute coronary events were reported, which may be related to the short-term use of β₂-agonists, the predominance of single-dose protocols, and the exclusion of patients with unstable cardiac conditions in most studies. Together, these findings suggest that administration of these medications is safe when no contraindications are present.

An important finding highlighted by Liou et al. was the presence of a subgroup of non-responders, with approximately one third of patients in the intravenous salbutamol group showing no significant reduction in serum potassium. From a clinical perspective, this lack of response may reflect the heterogeneity of patients with hyperkalemia, particularly those with advanced renal dysfunction, severe metabolic disturbances, or high baseline potassium levels, in whom transcellular potassium shifts alone may be insufficient to produce a measurable effect. Other contributing factors may include interindividual variability in β₂-adrenergic receptor sensitivity, the presence of concomitant acid–base disorders, or the use of medications that limit intracellular potassium uptake. In patients with chronic disease, functional desensitization of β₂-receptors may further attenuate the expected response. These observations reinforce the importance of recognizing that β₂-agonists are not uniformly effective across all clinical scenarios and should be considered as a supportive, temporizing intervention within a multimodal strategy for the management of hyperkalemia [15,32,33].

Several studies have evaluated the combination of β2-adrenergic agonists with insulin and glucose for the treatment of hyperkalemia, reporting results similar to those observed in the present investigation [9]. The administration of insulin with glucose has proven effective in lowering serum potassium due to its ability to promote potassium uptake into the intracellular space. However, this treatment carries a higher risk of hypoglycemia [33], requiring frequent and strict blood glucose monitoring. On the other hand, given the demonstrated efficacy of β2-adrenergic agonists when administered alone, and their lower need for monitoring, Emmanuel Montassier, in a letter to the editor [34], has suggested prioritizing these agents for the initial management of hyperkalemia in emergency settings, especially where intensive glycemic control may be complex or limited. This suggests that both therapeutic strategies may be effective; however, β₂-agonists may represent a more practical initial option in certain clinical scenarios where resources for close monitoring and prevention of hypoglycemia are limited. Nevertheless, caution is warranted, as it has been reported that approximately 20–40% of patients receiving nebulized β₂-agonists experience a reduction in serum potassium of less than 0.5 mEq/L [35]. In such contexts, the use of complementary strategies may be necessary and could potentially yield better clinical outcomes.

It is important to note that the study by Karuna Sree et al. included patients with hyperkalemia in an acute clinical context, consistent with acute kidney injury (AKI). This clarification is relevant because the therapeutic response to β₂-agonists may differ depending on whether hyperkalemia occurs in the setting of AKI or chronic kidney disease (CKD). Nevertheless, the study’s findings show that the expected potassium-lowering response is also observed in an acute scenario, supporting the use of β₂-agonists beyond populations with chronic or recurrent hyperkalemia and positioning them as a particularly valuable option in settings where immediate access to advanced interventions is not feasible.

Among the strengths identified in this review are the rigorous methodology employed, including a clearly defined systematic search strategy and a thorough risk of bias assessment for each included study, which contribute to the robustness, transparency, and reliability of the presented results.

Nevertheless, several limitations should be acknowledged. Most of the reviewed studies involved relatively small sample sizes and short follow-up periods, which hinder definitive conclusions regarding the duration of the potassium-lowering effect and the optimal dosing frequency when repeat administration is needed. Additionally, the lack of research in patients with serious comorbidities, especially cardiovascular or pulmonary disease, limits the generalizability of the results to more vulnerable populations. Therefore, the findings should be interpreted with caution in these clinical contexts. Future research should involve larger sample sizes and longer follow-up, with particular focus on trials in non-dialysis emergency populations, determination of optimal dosing, direct comparisons with insulin/glucose regimens, and comprehensive evaluations of cardiovascular safety, including the effects of higher doses in patients with concurrent cardiovascular conditions. Addressing these gaps will help improve the generalizability and clinical applicability of β₂-agonist therapy for hyperkalemia.

## Conclusion

The inhaled administration of β2-adrenergic agonists, particularly nebulized salbutamol, appears to reduce serum potassium acutely in adult patients with hyperkalemia, this therapy may be considered as a temporising measure in emergency situations, particularly where access to advanced treatments such as dialysis is limited. Careful monitoring for potential adverse effects, including tachycardia and hyperglycemia, is recommended. Further high-quality studies are needed to confirm these findings and better define optimal dosing, safety, and comparative efficacy.
